# A case of fungal keratitis and onychomycosis simultaneously infected by Trichophyton species

**DOI:** 10.1186/1471-2415-14-90

**Published:** 2014-07-11

**Authors:** Ki Won Jin, Hyun Sun Jeon, Joon Young Hyon, Won Rynag Wee, Wool Suh, Young Joo Shin

**Affiliations:** 1 Department of Ophthalomology, Hallym University College of Medicine, 948-1 Daerim1-dong, Youngdeungpo-gu, Seoul 150-950, Korea; 2 Department of Ophthalmology, Seoul National University College of Medicine, Seoul, Korea; 3 Department of Ophthalmology, Seoul National Bungdang Hospital, Seongnam, Gyeonggi-do, Korea

**Keywords:** Fungal keratitis, Trichophyton, Onychomycosis, Antifungal agents, Therapeutic keratoplasty

## Abstract

**Background:**

Fungal keratitis is difficult to treat that can result in corneal blindness requiring penetrating keratoplasty and in fungal endothalmitis. We report a case of fungal keratitis and onychomycosis simultaneously infected by Trichophyton.

**Case presentation:**

A 77-year old male presented with ocular pain, conjunctival injection, and severe loss of vision in his left eye. His best corrected visual acuity was hand movements in the left eye, and slit-lamp examination showed a corneal ulcer with feathery margin and hypopyon. Bacterial and fungal smear/culture showed no organism, and there was no improvement in spite of treatment with topical fortified 5% cefazolin and 2% tobramycin. Trichophyton species was identified by repeated cultures. We found onychomycosis on the patient’s foot, where the same fungal species were identified. Regimen was changed to topical itraconazole and systemic intravenous itraconazole. No clinical improvement was observed, so therapeutic penetrating keratoplasty and cryotherapy was done with continuation of antifungal therapy. The graft was clear at postoperative 1 month and no evidence of recurrence was found.

**Conclusion:**

It is important to identify the pathogen of keratitis because early identification of pathogen causing keratitis provides the appropriate treatment in early phase of keratitis. It is necessary to search for other fungal skin infections such as onychomycosis and athelete’s foot considering the fungal keratitis following skin infection. In addition, fungal skin infection including onychomycosis should be treated for prevention of fungal keratitis as soon as possible.

## Background

Fungal keratitis has been reported to be infected by fungi that are present on the surface of the eye as normal flora [1]. Fungal keratitis is often refractory to the anti-fungal treatment. Fungal keratitis, which is difficult to treat, can result in corneal blindness requiring penetrating keratoplasty and in fungal endothalmitis [2]. It is important to identify the pathogen of keratitis [1,2]. The most common pathogens of fungal keratitis are Candida albicans and Fusarium species [2], although the prevalent species differ in different geographical areas of the world [1]. Trichophyton is a wide distributed species of dermatophyte and a common pathogen of tinea capitis, flavus, and onychomycosis in dermatologic area [3,4], even though Trichophyton keratitis is not common [5,6]. Early identification of pathogen causing keratitis provides the appropriate treatment in early phase of keratitis, resulting in better prognosis. Recently, we experienced a case of fungal keratitis and onychomycosis simultaneously infected by Trichophyton. We report the Trichophyton keratitis associated with onychomycosis.

## Case presentation

**Figure 1:**
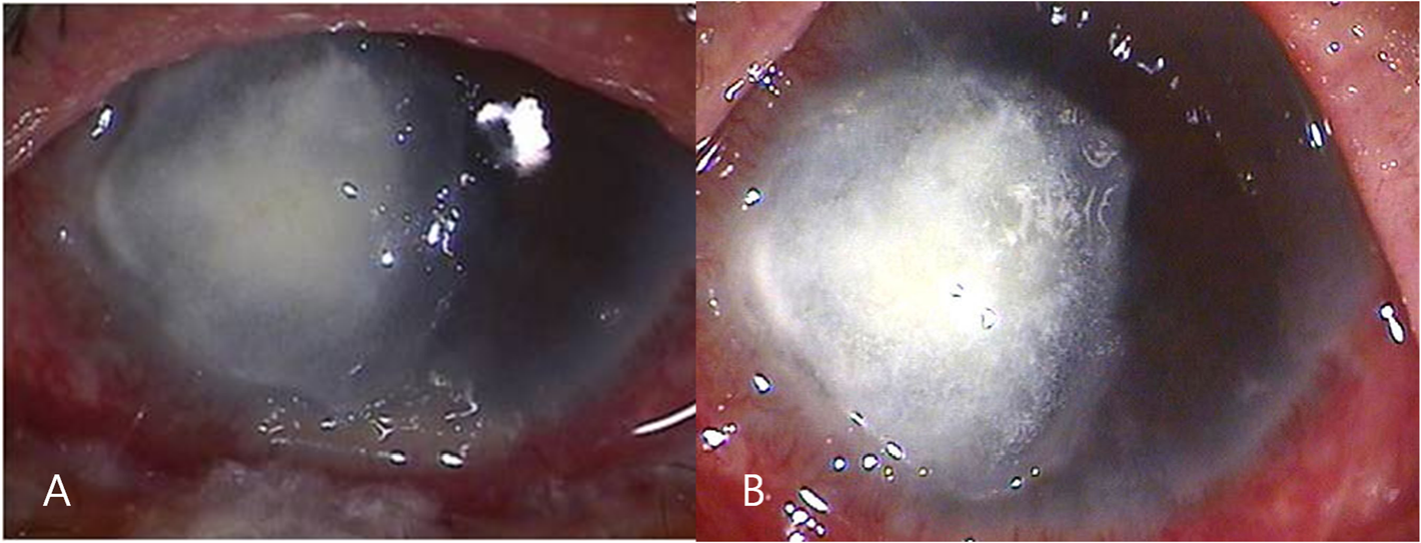
**Anterior photograph taken at the first visit. (A)** Slit-lamp examination showed a corneal epithelial defect, stromal infiltration (5 x 4 mm) with feathery margin, hypopyon and retrocorneal endothelial plaque. **(B)** The corneal infiltrate worsened although regimen was changed to topical 1% itraconazole and systemic itraconazole intravenously.

**Figure 2:**
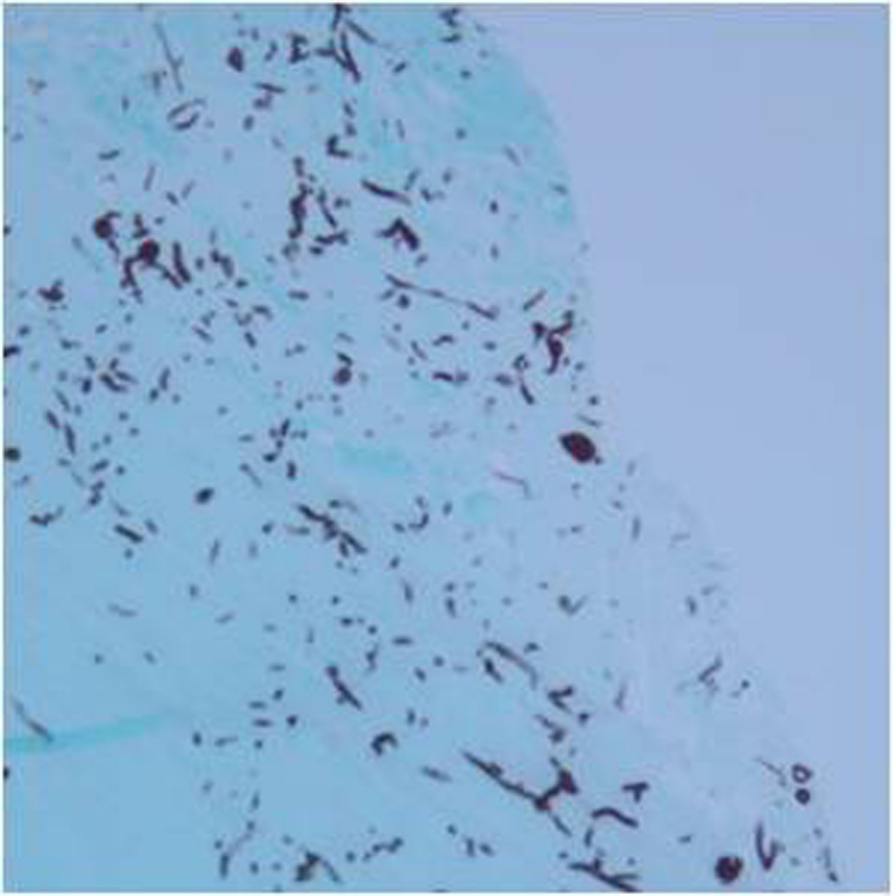
Histological examination showed many fungal hyphae and yeast forms in severe chronic active inflammation with methenamine silver nitrate staining, magnification x 40.

**Figure 3:**
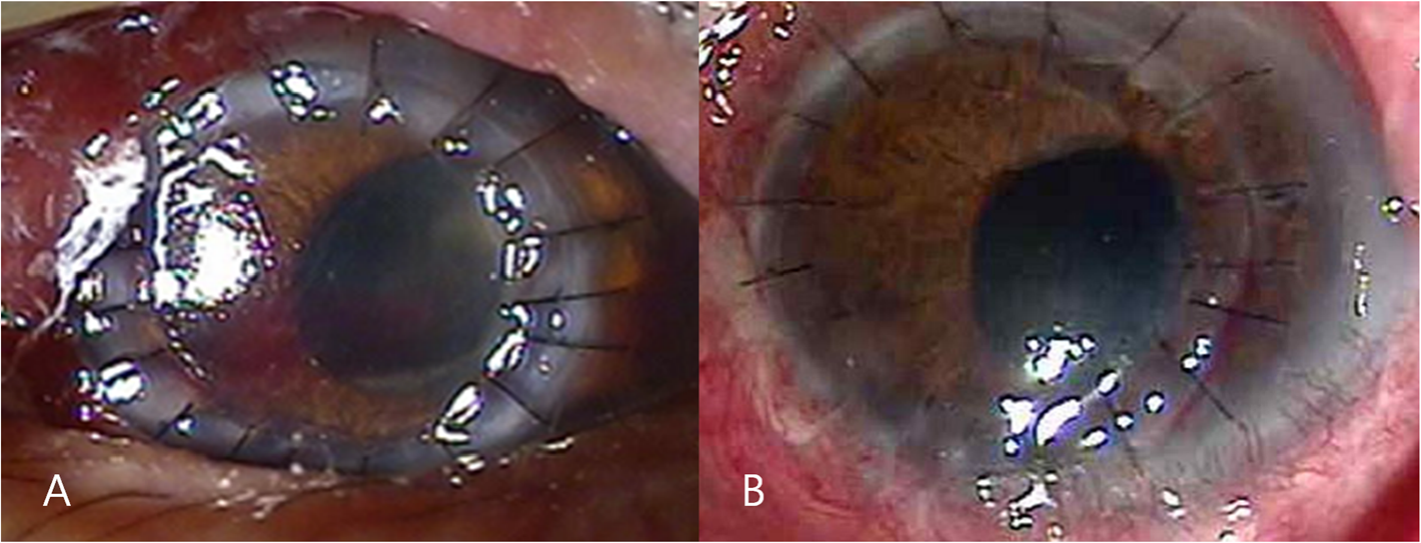
**Anterior photograph taken 4 days and 1 month after penetrating keratoplasty. (A)** The corneal graft was clear with moderate cellular inflammatory reaction in the anterior chamber after postoperative 4 days. **(B)** The graft was clear with no anterior chamber cellular reaction at postoperative 1 month.

A 77-year old male was referred to our clinic with ocular pain, conjunctival injection, and severe loss of vision in his left eye. His eye symptoms had started 3 weeks ago spontaneously. He had been treated with topical fortified 5% cefazolin and 2% tobramycin alternately 4 times a day for 4 days, but symptoms had not improved after treatment. He had a history of type II diabetes mellitus for 8 years, hypertension, chronic obstructive pulmonary disease, and pneumothorax. He underwent cataract surgeries in both of his eyes. At the first visit, his best corrected visual acuity (BCVA) was 20/40 in the right eye and hand movements in the left eye. The intraocular pressure was 20 mmHg in the left eye. Slit-lamp examination showed a corneal epithelial defect, stromal infiltration (5 × 4 mm) with feathery margins and hypopyon (Figure 1A). Corneal scrapings were taken immediately for smears and culture. Because the lesions progressed in spite of the fortified 5% cefazolin and 2% tobramycin, topical 1% voriconazole was administered every 2 hours, and itraconazole 200 mg orally twice a day was prescribed. Intrastromal and intracameral injection of voriconazole (50 μg/0.1 mL) was performed total 5 times every 4 days.Fungal hyphae were observed in KOH-stained corneal specimen stained on microscopic examination. Fungal culture on Sabouraud dextrose agar identified Trichophyton species after 7 days of incubation. We inspected the patient’s body for other fungal skin infection, and found onychomycosis on the patient’s foot. The patient had suffered from foot onychomycosis for 10 years. Trichophyton, which was the same species causing the fungal keratitis, was identified on culture of nail. The treatment regimen was changed to topical 1% itraconazole every 2 hours and 200 mg of systemic itraconazole intravenously every 12 hours. In spite of the treatment, no clinical improvement was observed for 4 days and the corneal infiltrate worsened (Figure 1B). Therefore, therapeutic penetrating keratoplasty and cryotherapy was done. Many fungal hyphae and yeast forms in severe chronic active inflammation was found in the surgical specimens under microscopic examination with methenamine silver nitrate (MSN) staining (Figure 2). Topical natamycin per 2 hours, moxifloxacin per 6 hours, and oral terbinafine (250 mg, once a day) was prescribed for postoperative management.Four days after the penetrating keratoplasty, the corneal graft was clear with moderate cellular inflammatory reaction in the anterior chamber (Figure 3A). Patient was discharged and treated with topical natamycin and oral terbinafine for 8 weeks after surgery. The graft was clear with no anterior chamber cellular reaction at postoperative 1 month (Figure 3B). His visual acuity was “counting finger” at 50 cm distance in the right eye and there was no evidence of recurrence.

## Conclusions

Fungal keratitis is difficult to diagnose and treat because fungi often proliferate rapidly, cause severe stromal necrosis and enter the anterior chamber by penetrating an intact Descemet’s membrane [7,8]. In this case, we report the first case of keratitis and onychomycosis infected by Trichophyton species simultaneously. Although Mohammad et al. reported 5 cases of Trichophyton fungal keratitis, none of the patients has been reported to have the concurrent mycotic skin diseases [5]. The ocular involvement can be caused by spreading from an adjacent skin lesion or from a distant infected source outside the patient [8]. In this case, the patient had suffered from onychomycosis in his foot for 10 years. The organism of his onychomycosis was Trichophyton, which was the same organism causing keratitis. His keratitis can be the secondary infection from his onychomycosis. A case of simultaneous infection of fungal keratitis and onychomycosis by cryptococcus laurentii has been reported and the spread of cryptococcus laurentii from onychomycosis has been suggested [9].

Trichophyton species has been reported to secrete collagenase specific for human-native collagen and gelatin [10]. Trichophyton species have dozens subtypes although T. rubrum and T. mentagrophytes are common pathogens of onychomycosis [11]. In this case, the type of Trichophyton species was not identified. Through the expression an extracellular collagenase with keratinolytic potential, Trichophyton species can cause a severe stromalysis leading to the loss of vision [5,10]. Rapid diagnosis and aggressive medical treatment is of most importance to preserve the vision and have better therapeutic results [1,5]. Shenoy et al. [8] reported a case of Trichophyton keratitis that was not responding to 5% natamycin and treated with topical 2% fluconazole. Mohammad et al. [5] reported 5 cases that were sensitive to topical natamycin although they did not respond to both amphotericin B and miconazole. Our case did not respond to topical natamycin and oral terbinafine. Thus, the patient underwent penetrating keratoplasty and fungal keratitis did not recur.

In conclusion, it is important to identify the pathogen of keratitis because early identification of pathogen causing keratitis provides the appropriate treatment in early phase of keratitis. It is necessary to search for other fungal skin infections such as onychomycosis and athelete’s foot considering the fungal keratitis following skin infection. In addition, fungal skin infection including onychomycosis should be treated for prevention of fungal keratitis as soon as possible.

### Consent

Written informed consent was obtained from the patient for publication of this case report and any accompanying images.

## Competing interests

The authors declare that they have no competing interests.

## Authors’ contributions

KWJ and YJS were responsible for the conception and design of the project. YJS and WS were responsible for acquisition and interpretation of data. KWJ was responsible for drafting the article. JYH and HSJ revised the manuscript critically for important intellectual content. All authors approved the final version to be published.

## Pre-publication history

The pre-publication history for this paper can be accessed here:

http://www.biomedcentral.com/1471-2415/14/90/prepub
